# Human SCARB2-Mediated Entry and Endocytosis of EV71

**DOI:** 10.1371/journal.pone.0030507

**Published:** 2012-01-17

**Authors:** Yi-Wen Lin, Hsiang-Yin Lin, Yueh-Liang Tsou, Ebenezer Chitra, Kuang-Nan Hsiao, Hsiao-Yun Shao, Chia-Chyi Liu, Charles Sia, Pele Chong, Yen-Hung Chow

**Affiliations:** 1 National Institutes of Infectious Disease and Vaccinology, National Health Research Institutes, Taiwan, Republic of China; 2 Graduate Program of Biotechnology in Medicine, Institute of Molecular Medicine, National Tsing Hua University, Hsinchu, Taiwan; 3 Graduate School of Life Science, National Defense Medical Center, Taipei, Taiwan; 4 Graduate Institute of Immunology, China Medical University, Taichung, Taiwan; Agency for Science, Technology and Research - Singapore Immunology Network, Singapore

## Abstract

Enterovirus (EV) 71 infection is known to cause hand-foot-and-mouth disease (HFMD) and in severe cases, induces neurological disorders culminating in fatality. An outbreak of EV71 in South East Asia in 1997 affected over 120,000 people and caused neurological disorders in a few individuals. The control of EV71 infection through public health interventions remains minimal and treatments are only symptomatic. Recently, human scavenger receptor class B, member 2 (SCARB2) has been reported to be a cellular receptor of EV71. We expressed human SCARB2 gene in NIH3T3 cells (3T3-SCARB2) to study the mechanisms of EV71 entry and infection. We demonstrated that human SCARB2 serves as a cellular receptor for EV71 entry. Disruption of expression of SCARB2 using siRNAs can interfere EV71 infection and subsequent inhibit the expression of viral capsid proteins in RD and 3T3-SCARB2 but not Vero cells. SiRNAs specific to clathrin or dynamin or chemical inhibitor of clathrin-mediated endocytosis were all capable of interfering with the entry of EV71 into 3T3-SCARB2 cells. On the other hand, caveolin specific siRNA or inhibitors of caveolae-mediated endocytosis had no effect, confirming that only clathrin-mediated pathway was involved in EV71 infection. Endocytosis of EV71 was also found to be pH-dependent requiring endosomal acidification and also required intact membrane cholesterol. In summary, the mechanism of EV71 entry through SCARB2 as the receptor for attachment, and its cellular entry is through a clathrin-mediated and pH-dependent endocytic pathway. This study on the receptor and endocytic mechanisms of EV71 infection is useful for the development of effective medications and prophylactic treatment against the enterovirus.

## Introduction

Enterovirus 71 (EV71), a positive single stranded RNA virus belonging to the family of *Picornaviridae* is known to manifest hand-foot-and mouth disease (HFMD) in young children, and can lead to the development of severe neurological diseases including aseptic meningitis, cerebellar encephalitis, and acute flaccid paralysis culminating in fatality in some patients especially children [1], [2], [3], [4], [5]. The first EV71 infection was recorded in California in 1969 [6], and then opened an outbreak in Bulgaria in 1975 resulted in 44 fatal cases [7], also reported 47 fatalities in Hungary in 1978 [8], 34 fatalities in Malaysia in 1997 [1], [9], 78 fatalities in Taiwan in 1998 [10], [11]. Following epidemics in Taiwan that caused 25 deaths in 2000, 26 deaths in 2001, and 14 deaths in 2008 [12], [13], [14], and more cases of EV71 infection are sporadically reported in many countries. Till now the treatment and control of EV71 infection are only symptomatic due to the lack of effective medications and unavailability of a prophylactic vaccine [15].

Two different membrane proteins, human P-selectin glycoprotein ligand-1 (PSGL-1; CD162) [16] and human scavenger receptor class B, member 2 (SCARB2) [17] have been identified as cellular receptors for EV71. PSGL-1 is a sialomucin membrane protein widely expressed in leukocytes that plays a role in early stages of inflammation [18], [19], [20]. SCARB2, also called lysosomal integral membrane protein 2 or CD36b like-2, has two transmembrane domains with the N and C termini located in the cytosol [21]. It is expressed in many tissues, participates in membrane transport and the reorganization of the endosomal/lysosomal compartment [22] and high density lipoprotein endocytosis (HDL) [23]. Based on their PSGL-1-binding capability, different EV71 isolates are classified into PSGL-1-dependent and PSGL-1-independent strains. SK-EV006 (genogroup B3), C7/Osaka (B4), KED005 (C1), 1095 (C2) and 75-Yamagata (C4) are PSGL-1-dependent strains. BrCr (genogroup A), Nagoya (B1), and 02363 (C1) are PSGL-1-independent isolates [16], [24]. Some EV71 strains such as BrCr (A), Nagoya (B1), and Isehara (C) have been reported to utilize SCARB2 as a receptor. Expression of human SCARB2 receptor in unsusceptible cell lines was found to facilitate these strains of EV71 and coxsackievirus A16 (CVA16) infection resulting in the development of cytopathic effects [17].

Viruses enter into host cells through endocytosis followed fusion of the viral envelope with the endosomal membrane facilitating delivery of the viral genome into the cytosol. Although the endocytic pathways used by some viruses, such as influenza virus [25], hepatitis C virus (HCV) [26], respiratory syncytia virus (RSV) [27], [28], severe acute respiratory syndrome coronavirus (SARS-CoV) [29], vesicular stomatitis virus (VSV) [30], [31], simian virus 40 (SV40) [32], Ebola virus [33], and echovirus I [34] have been determined. Viruses entering cells through clathrin- or caveolin-mediated endocytosis, as well as clathrin- and caveolin-independent pathways have been reported [35], [36], [37]. In clathrin-mediated endocytosis, virus-bound receptors are targeted to clathrin-coated pits (CCPs), which mature into clathrin coated vesicles (CCVs) resulting in the internalization of viruses with their receptors. Caveolin-mediated endocytosis is also a well-characterized process, which involves the association of glycolipid rafts of plasma membrane with caveolins resulting in their internalization [37]. Dynamins, the large GTPase of molecular size 100 KDa are involved in the budding and scission of nascent vesicles from the parent membranes during clathrin-mediated endocytosis [38], [39]. Several studies have identified the recruitment of dynamin to the neck of nascent CCVs prior to fission [40], [41], [42]. Additionally, dynamins have been implicated in the budding of caveolin, phagocytosis and vesicle cycling at the neuron-muscular junction [43]. Recent study has mapped the encoding sequence at the region of amino acid 142 to 204 of human SCARB2 that is critical for EV71 binding and infection [44]. The clathrin-mediated endocytosis as the entry pathway for EV71 infection was identified [45]. However, the detail of receptor-mediated entry mechanism for EV71 has still needs more exploration.

Our investigation focuses on the entry mechanism of EV71 in SCARB2-expressing cells. We have infected the SCARB2-overexpressing NIH3T3 cells (3T3-SCARB2), RD, and Vero cells with EV71/E59 strain, a B4 sub-genotype isolated in Taiwan in 2002. Expression of SCARB2 had made the 3T3-SCARB2 and RD cells susceptible to EV71 infection, proving that SCARB2 is a functional receptor of EV71. Both viral infection and replication was well supported in 3T3-SCARB2 and RD cells, while SCARB2-independent EV71 susceptibility was observed in Vero cells. We demonstrates that EV71 entry into 3T3-SCARB2 is through clathrin-dependent endocytosis which was inhibited significantly by knocking down the clathrin or dynamin expression using specific siRNAs or by using inhibitors of clathrin-dependent endocytosis. Moreover, the examined endocytosis of EV71 is pH-dependent as it was inhibited by endosomal acidification inhibitors. Taken together, our results demonstrate that EV71 binds to SCARB2 and triggers a clathrin- and dynamin-dependent endocytosis for its entry.

## Materials and Methods

### Cell culture

African green monkey kidney (Vero) (ATCC No. CCL-81) and human rhabdomyosarcoma (RD) (ATCC No. CCL-136) cells were provided by the Taiwan Centers of Disease Control (Taiwan CDC); the original cell lines were obtained from the American Type Culture Collection (ATCC). A549 (ATCC No. CCL-185), a human lung adenocarcinoma lines, were purchased from ATCC. NIH3T3 mouse fibroblast cells (ATCC No. CRL-1658) were kindly obtained from Dr. Pele Chong, National Institutes of Infectious Disease and Vaccinology, National Health Research Institutes, Taiwan. The original NIH3T3 cell lines were purchased from ATCC. Vero cells were cultured in VP-SFM medium (GIBCO) supplemented with 5% fetal bovine serum (FBS) (Biological Industries), and 1% penicillin/streptomycin (P/S) (Biological Industries). NIH3T3, RD and A549 cells were grown and maintained in Dulbecco's modified Eagle's medium (Hyclone) plus 10% FBS, and 1% P/S. Cells were maintained in an incubator at 37^o^C and equilibrated with 5% CO_2_.

NIH3T3 cells at 80% confluency were transfected with pCMV-SCARB2 plasmid by cationic polymer-DNA transfection method. NIH3T3 cells were grown in a 12-well plate, and 0.5 µg of plasmid DNA pre-mixed with 1 µL of TurboFect transfection reagent (Fermentas) was added to 50 µL culture medium for each well and then cultured in the incubator for 24 hours. Stable cells overexpressing SCARB2 were selected in the presence of 800 µg/ml of G418 (Sigma-Aldrich). Stable cell line 3T3-SCARB2 was established by expansion of a single cell originally cultured in 96-well plate in the presence of G418 after two weeks of culture.

### Plasmids and viruses

Human SCARB2 gene sequence published recently [17] was codon-optimized and synthesized commercially by GENEART, Germany and inserted into pcDNA3.1(-) vector (Invitrogen) containing enhancer/promoter of cytomegarovirus (CMV), simian virus 40 (SV40) poly A tail, and neomycin-resistant gene, at EcoRI/BamHI cloning site to obtain pCMV-SCARB2 plasmid.

EV71/E59 strain, a B4 sub-strain isolated in Taiwan in 2002 by Taiwan CDC was propagated in Vero or 3T3-SCARB2 cells. Briefly, 2×10^6^ cells cultured in a 10-cm dish were infected with live EV71/E59 virus at a MOI (multiplicities of infection) of 1×10^−3^. The culture supernatant was harvested 5 days after infection and passed through a 0.45 µm filter (Sartorius) to remove the cell debris. The culture supernatant was tested in a standard virus plaque forming assay to determine its infectious viral content. It was aliquoted and stored at −80°C as the viral stock. Recombinant lentivirus-eGFP which carries green fluorescence protein (GFP) was obtained from the National RNAi Core Lab in Genomics Research Center of Academia Sinica, Taiwan.

### Flow cytometry

1×10^6^ NIH3T3, 3T3-SCARB2, Vero, and RD cells were harvested from trypsin-free dissociation buffer (GIBCO) treated culture dishes and spun down to remove the supernatant. Cells were washed once with 1×PBS (pH = 7.4) and then fixed with permealization/fixation solution (70% ice-cold methanol in PBS) or non-permealization/fixation solution (0.5% paraformalydehyde in PBS) for overnight. The cells were washed, and 1 mL of 1 to 1000 dilution of biotinylated anti-SCARB2 antibody (R&D) was added and incubated at 4°C for 1 hour. After incubation, the cells were washed and stained with 1∶2000 diluted fluorescence isothiocyanate (FITC)-conjugated streptavidin (Biolegend) for 30 minutes. SCARB2-expressing cells were detected using FACScan flow cytometer and analyzed using the CellQuest software (Becton Dickinson Immunocytometry System). 3T3-SCARB2 cells stained only with FITC-streptavidin were used as conjugate control for deducting the background.

### EV71- specific enzyme-linked immunosorbent assays (ELISA)

Cells cultured at a density of 10^4^ per well in 96-well plate were incubated with varied MOIs of EV71 in 100 µL of serum-free DMEM medium for one hour at 37°C. After one hour of infection, the cells were washed three times with 1×PBS (pH = 7.2), and cultured in 1serum-free DMEM for another 24 hours. Cells were washed again with 1×PBS (pH = 7.2) and then fixed with 100 µL of 100% methanol for 30 minutes at room temperature, and then 250 µL of 5% skim milk-PBS was added to each well to block the non-specific binding of the detection antibodies. 100 µL of the antibody solution (containing 0.1 µL of the E1 monoclonal antibody, produced from a hybridoma line generated from EV71/E59-immunized BALB/c mouse at National Health Research Institutes, vaccine Center, Taiwan) prepared in PBS containing 5% skim milk was added to each test well to detect the viral capsid proteins. After 2 hours incubation at room temperature, the plate was washed four times by pipeting 250 µL of 1x PBS into the individual wells. 100 µL of HRP-conjugated anti-mouse IgG (Jackson ImmunoResearch) diluted at 1 in 5,000 in assay buffer was then added to each well and incubated for I hour at room temperature before washing the plate four times with 1x PBS. 70 µL of TMB peroxidase substrate (SureBlue™, KPL) was added to the individual assay well and allowed to react for 30 min at room temperature. The reaction was stopped by adding 70 µL 2N H_2_SO_4_ to each well and the absorbance was recorded at OD 450 nm using an ELISA reader (Spectra Max M2 model, Molecular Devices, USA).

### Antibodies and western blot

Anti-VP2 specific monoclonal antibody, MAB979, was obtained from Millipore-Chemicon International. Anti-SCARB2 antibody was purchased from R&D. Antibodies specific to clathrin and dynamin were purchased from Abcam and Santa Cruz Biotechnology, Inc., respectively. Anti-caveolin-1 antibody was purchased from Cell signaling. *De novo* synthesis of VP1 and VP2 in EV71-infected cells and expression of SCARB2 in the cells were assessed by Western blot.

Cell lysates were prepared by treatment of 1−2×10^6^ cells in 100 µL of ice-cold lysis buffer (0.5% sodium deoxycholate, 0.1% sodium dodecyl sulfate (SDS), 0.5% NP-40, 50 mM TRIS, 150 mM NaCl) with the addition of a protease inhibitors cocktail (Roche) and 1 mM PMSF (Sigma-Aldrich). Lysates were then centrifuged for 20 min at 10,000 rpm, at 4^o^C to sediment cell debris. Cell lysate was subjected to SDS-polyacrylamide gel electrophoresis (SDS-PAGE) (Amersham Biosciences). This entailed loading 10 µg of the cell lysate mixed with loading dye per well of a 10% SDS-PAGE. Following electrophoresis at 100 V for 90 minutes in 1X Tris-glycine SDS-running buffer, the resolved proteins were transferred onto a nitrocellulose membrane (Hybond-ECL, Amersham Biosciences). Protein-containing membrane was soaked in 5% skim milk in PBS, pH 7.4 for 30 min at room temperature, then washed three times with 10 mL of assay buffer [PBS, pH 7.4 containing 0.05% Tween 20]. The membrane was incubated with 1: 5000 diluted MAB979, or 1: 2000 diluted anti-SCARB2 antibody, for 14–16 hours at 4°C, and subsequently washed 5 times with 15 mL of assay buffer followed by incubation with horse radish peroxidase (HRP) conjugated donkey anti-mouse secondary antibody (Jackson ImmunoResearch) at a dilution of 1 in 5,000 (for MAB979), or HRP-conjugated anti-goat secondary antibody (Jackson ImmunoResearch) at a dilution of 1∶5,000 (for anti-SCARB2 antibody). After 1 hour incubation at room temperature, the membrane was washed 5 times with the assay buffer, and 1 mL of Super Signal West Pico chemiluminescent substrate (Pierce) was then layered onto the membrane. The reaction was detected by exposing the membrane to an X ray film (Kodak) at 30 sec, 1, 3, and 5 minutes intervals. When necessary membranes were stripped with restoring buffer (Pierce) and used again with another antibody.

### Co-Immunoprecipitation

400 µg of cell lysates from 3T3-SCARB2 and parental 3T3 cells were mixed with 3×10^6^ pfu of EV71 and incubated at 4°C for 1 h with occasionally rocking. Following lysate-viruses mixtures was rocked with 20 µL of protein G agarose beads (Santa Cruz Biotechnology) and 1 µg of anti-SCARB2 antibody at 4°C for 3.5 hours. After five washes with lysis buffer, beads were resuspended in 50 µL SDS loading buffer, boiled for 5 min and then 10 uL of samples were taken and subjected to SDS-PAGE and western blotting with anti-EV71 MAB 979 antibody. Detection was achieved by using appropriate secondary antibodies labeled with horseradish peroxidase as described above. Each experiment has been repeated at least two times independently.

### Immune plaque forming assay

The assay was carried out by seeding 1.5×10^5^ NIH3T3, 3T3-SCARB2, Vero, or RD cells in individual wells of a 12-well tissue culture plate (Corning). 500 µL of 10^3^ to 10^8^ dilutions of EV71/E59 in serum-free culture medium was then added to each well followed by one hour incubation at 35^o^C. 1 mL of 2.0% methyl cellulose in DMEM was overlaid onto the cells. The cultures were kept in a 35^o^C incubator equilibrated with 5% CO_2_ for 5 days. Development of plaques was detected by removing the supernatant, washing the cells once with cold PBS, and treating them with 0.5 mL of 2.0% paraformaldehyde (Merck). Fixation was allowed by incubating the plate at 4^o^C for 10 minutes. Fixation buffer was removed and 250 µL of assay buffer containing 1 in 5,000 dilution of the E1 monoclonal antibody was then added to the individual test well. The assay plates were incubated for 1 hour at 37^o^C for E1 antibodies to bind to the viral VP1 capsid protein, expressed on EV71-infected cells. Individual test wells were then washed 3 times with 0.5 mL of assay buffer. 100 µL of an anti-mouse IgG-HRP (horse radish peroxidase) conjugated antiserum (Jackson ImmunoResearch) at 1 in 5,000 dilution prepared in assay buffer was added to each test wells. After leaving the plates for 1 hour incubation at room temperature, the test wells were washed three times with the assay buffer. The plates were blotted dry on paper towel, 100 µL of TMB substrate (KPL) was added to the individual assay wells and then the plates were incubated in dark for 30 minutes. The plaques representing cells infected by EV71 virus were seen as black spots in each test well and their number was taken for calculating plaque-forming units (pfu) to represent the virus infectivity.

### Down-regulation of cellular gene expression by siRNA

3T3-SCARB2 cells at 80% confluency were transfected with oligonucleotides of siRNA specific to target gene or the control siRNA by liposome-oligonucleotide transfection method. Briefly, 50 µL culture medium containing 50 or 100 pmoles of siRNA pre-mixed with 1 µL of TurboFect siRNA transfection Reagent (Fermentas) was added per well of a 12-well plate and incubated at 37^o^C for 24 or 48 hours in an incubator. After incubation, the cells were infected with EV71 (MOI = 0.04) in serum-free culture medium followed by 1 hour incubation at 37^o^C before washing three times with serum-free culture medium. After 24 or 48 hours of incubation, cells lysates were prepared for western blot. SCARB2 siRNA (sc-41546) was purchased from Santa Cruz Biotechnology, Inc. SiRNAs specific to mouse clathrin light chain B (Gene bank Accession No. M20469) (One-TARGET plus SMART pool, Cat. No. L-046978-01-0005), mouse dynamin 2 (Gene bank Accession No. L36983) (One-TARGET plus SMART pool, Cat. No. L-044919-02-0005), mouse caveolin-1 siRNA (Invitrogen), and negative control siRNA were purchased from Invitrogen. The blots were quantified using Image –Pro Plus 6.0software.

### Inhibition of endocytosis and endosomal acidification

1×10^5^ 3T3-SCARB2 cells seeded in a 12-well plate one day prior to the experiments were treated with various concentrations of inhibitor for one hour and then infected with EV71 (MOI = 0.04) in serum-free culture medium for 1 hour at 37°C in the presence of inhibitor. The cells were washed three times with serum-containing culture medium and after additional 24 hours of culturing in the presence of the inhibitor, the cells were harvested and lysed for immunoblotting using MAB979 antibody. For lentivirus-eGFP infection, 4×10^5^ A549 cells were seeded in a 12-well plate one day prior to the treatment with genistein (dissolved in 0.1% DMSO) or vehicle alone for one hour and then infected with lentivirus-eGFP (MOI = 0.1). After infection, cells were washed and then following cell culture until 72 hours. Without infection with lentivirus-eGFP of A549 cells were included as GFP negative control. The same field of fluorescent and visible pictures was taken under the *UV*-fluorescent microscopy (Olympus DP70) and GFP-positive cells were counted. For the assay of the internalization of cholera toxin subunit B (CT-B), 1×10^5^ 3T3-SCARB2 cells were grew on a coverslip one day and then washed to remove any serum prior to the treatment with filipin (dissolved in 0.1% DMSO vehicle) or vehicle alone for one hour at 37^o^C. All subsequent incubations contained the inhibitors. Cells were cooled to 15^o^C and then incubated with 5 µg/mL alexa fluor 594 CT-B conjugate (dissolved in 0.1% DMSO) (Invitrogen). After 30 minutes incubation, cells were washed and either fixed immediately. The cells were fixed with 4% paraformaldehyde for 20 minutes at 37^o^C and washed three times in PBS. The slides were then mounted with a coverslip and the cells observed in the same field of fluorescent and visible pictures was taken by the confocal microscopy (Leica TCS SP5 II).

Depletion of cholesterol from the plasma membrane using MβCD was done as described [46]. Cells were treated with serial dilutions of MβCD for 30 minutes prior to the infection with EV71 as described above.

The following compounds used as inhibitors were purchased from Sigma-Aldrich, St Louis, MO, USA: genistein, filipin, methyl β-cyclodextrin (MβCD), chloroquine, and ammonium chloride (NH_4_Cl). chlorpromazine (CPZ) was obtained from Alexis Biochemicals.

## Results

### Expression of SCARB2 in transfected NIH3T3 cells

To explore the role of SCARB2 while EV71 virus entry, EV71 virus was cultivated in RD and Vero cells that already express SCARB2 and in NIH3T3 cells engineered to express human SCARB2. Human SCARB2 gene was cloned into pCMV expression vector under the CMV promoter to obtain pCMV-SCARB2 plasmid. NIH3T3 cells which are naturally not susceptible to EV71 were transfected with pCMV-SCARB2 plasmid and selected with G418 to establish a stable SCARB2-expressing cell line, 3T3-SCARB2.

**Figure 1:**
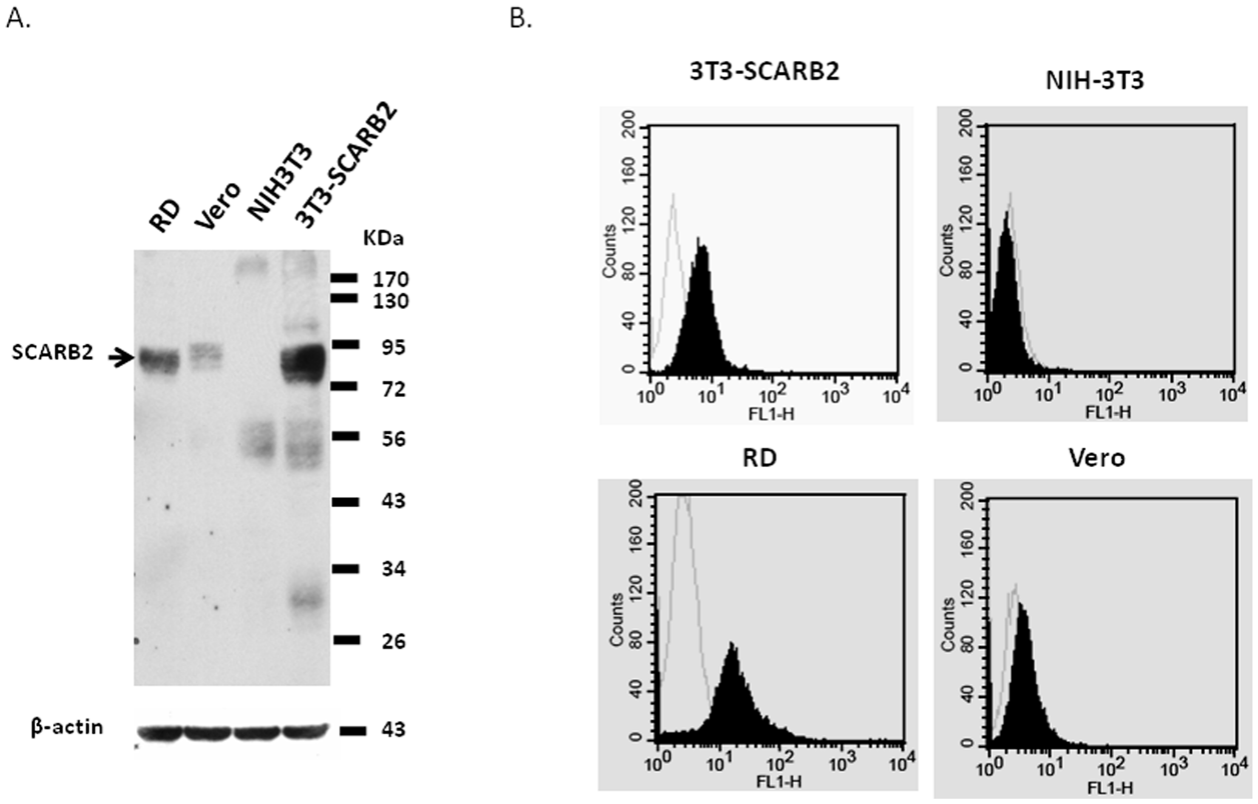
Expression of SCARB2 in RD, Vero, NIH3T3, and 3T3-SCARB2 cells. (A) Lysates prepared from the tested cells were analyzed by immunoblotting using a polyclonal rabbit anti-SCARB2 antiserum. The SCARB2 protein has molecular size of 80 KDa. The internal cellular β-actin was detected by blotting the stripped membrane with monoclonal anti-β-actin antibody which corresponded to 44 KDa products. (B) The tested cells were incubated with biotinylated anti-SCARB2 antiserum (filled curve) or without antiserum (empty curve) prior fixed by methanol and then stained with FITC-conjugated avidin. Stained cells were run on a FACScan flow cytometer and analyzed by using CellQuest software (Becton Dickinson Immunocytometry System).

The expression levels of SCARB2 in Vero, RD and 3T3-SCARB2 cells were examined by Western blotting using a polyclonal antibody specifically against the cytosolic domain of SCARB2. The expressed SCARB2 protein was identified to have a molecular weight of 80 KDa and the determined expression level was very high in 3T3-SCARB2, moderate in RD cells, but very little in Vero cells. No SCARB2 was detected in the cell lysate from NIH3T3 cells (Fig. 1A). The following treatment of cells with permealization/fixation solution and then analyzed by flow cytometry also confirmed the cytosolic expression of SCARB2 in 3T3-SCARB2, RD, and Vero cells (Fig. 1B). However, flow analysis of NIH3T3, 3T3-SCARB2, RD, and Vero cells that pre-treated with a non-permealization/fixation solution to maintain the intact membrane structure could not detect SCARB2 on the surface (data not shown). It is probably due to SCARB2 dynamically cycling between the endosomal/lysosomal compartments and plasma membrane while transports cellular proteins from outside of cells into the cells [23]. Moreover, another identified EV71 receptor, PSGL-1, usually expressed in leukocytes [16]. Our RT-PCR analysis also revealed that NIH3T3, Vero and RD cells did not express PSGL-1 (data not shown).

### SCARB2 expression in 3T3-SCARB2 facilitates EV71 infection

**Figure 2:**
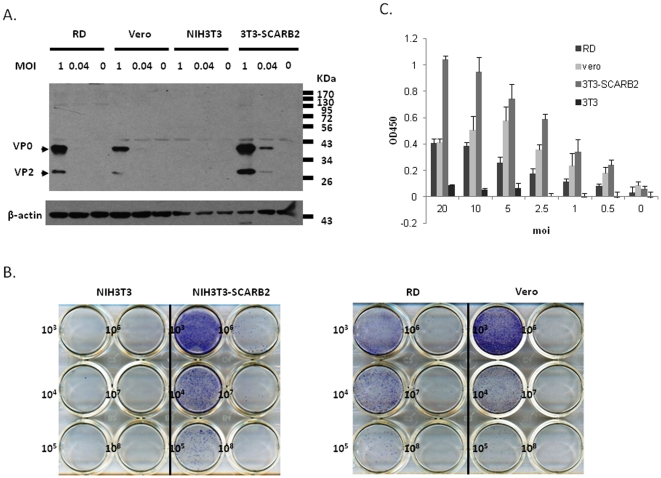
Susceptibility of SCARB2-expressing cells infected with EV71. (A) Four different cells, NIH3T3, 3T3-SCARB2, RD, and Vero cells, were infected with two different MOI of EV71 and then cultured for 48 hours. After incubation, cell lysates were prepared for western blot using anti-capsid MAB979 antibody. (B) Immune plaque forming assay was performed in four different cells infected with 10-fold serial diluted 10^8^ pfu/ml stocks of EV71 as described in the Materials and Methods. The immuno-stained plaques were counted and photography was taken. (C) NIH3T3 or SCARB2-expressing NIH3T3, RD, and Vero cells cultured in 96-well plate were inoculated with various MOI of EV71 for one hour at room temperature. After incubation, cells were washed three times with PBS and then cultured for another 24 hours before fixed by methanol. EV71 capsid protein in the cells was detected by ELISA assay as described in the materials and methods.

To confirm that SCARB2 expression *per se* facilitates infection with EV71 virus, 3T3-SCARB2, RD, Vero and NIH3T3 cells were inoculated with two concentrations of EV71 virus and examined for viral infection. Cytopathic effects were observed in 3T3-SCARB2, RD, and Vero cells. The expression of EV71 capsid proteins namely VP2 and VP4 in the infected cells was demonstrated by western blot using a VP2-specific monoclonal antibody MAB979 which has been identified [47]. Previous studies showed that multiple capsid proteins of VP0 (38 KDa, a precursor product of VP2+VP4), VP1 (36 KDa), VP2 (28 KDa), VP3 (25 KDa) and VP4 (8 KDa) were detected in the EV71-infected cells [48], [49], [50]. Corroborating these findings, we observed that 3T3-SCARB2, Vero and RD cells infected with EV71 were able to express capsid proteins with molecular weights in the range of 38 KDa (for VP0), and 28 KDa (for VP2), and 25 KDa (for VP3) (Fig. 2A). In NIH3T3 cells, neither the cytopathic effect nor the expression of viral capsid proteins was seen confirming that these cells were unsusceptible to EV71 infection and that SCARB2 expression had converted it into a susceptible cell line. The expression level of viral capsid protein was found to synchronize with the increase in multiplicity of infection (MOI) of EV71 from 0.04 to 1. The level of expression of viral capsid protein was the highest in 3T3-SCARB2 cells, moderate in RD cells and the lowest in Vero cells, in which were corresponding to the individual expression levels of SCARB2. This finding confirms a correlation between SCARB2 expression and susceptibility to EV71.

The correlationship was further investigated by infecting NIH3T3, 3T3-SCARB2, Vero, and RD cells with serial dilutions (10^−3^ to 10^−8^ pfu) of EV71 followed by determination of plaque formation by immuno-plaque assay. We observed that 3T3-SCARB2 with the highest level of SCARB2 expression (Fig. 1A) was susceptible to ∼1×10^−8^ dilution of EV71 whereas RD cells with moderate expression were susceptible to ∼5×10^−7^ dilution of EV71. Interestingly, Vero cells with the lowest level of SCARB2 expression acquired ∼3×10^−7^ dilution of EV71 to get infected (Fig. 2B), and as expected, no plaques were formed in NIH3T3 cells, which were unsusceptible. Moreover, ELISA assay to compare the cell susceptibility for EV71 infection was also performed (Fig. 2C) as described in the Materials and Methods. By using E1 antibody against EV71 VP1 [47], 3T3-SCARB2 cells expressed the highest EV71 capsid protein was detected in a dose-dependent manner. No EV71 capsid protein was detected in NIH3T3 cells which do not express SCARB2. RD cells showed the moderate expressed EV71 virus. Nevertheless, Vero cells could be efficiently infected with EV71 virus (Fig. 2C), in spite of their low levels of SCARB2 (Fig. 1A). By comparison of the order of the susceptibility to EV71 infection (3T3-SCARB2>Vero>RD) with the level of SCARB2 expression (3T3-SCARB2>RD>Vero), it is possible that Vero cells possess other binding proteins to support EV71 infection.

Our results not only confirm the role of SCARB2 as a receptor for EV71 infection but also demonstrate that the degree of susceptibility to EV71 depends on the expression levels of SCARB2 in the cells.

### Interaction of EV71 capsid protein with SCARB2 receptor

**Figure 3:**
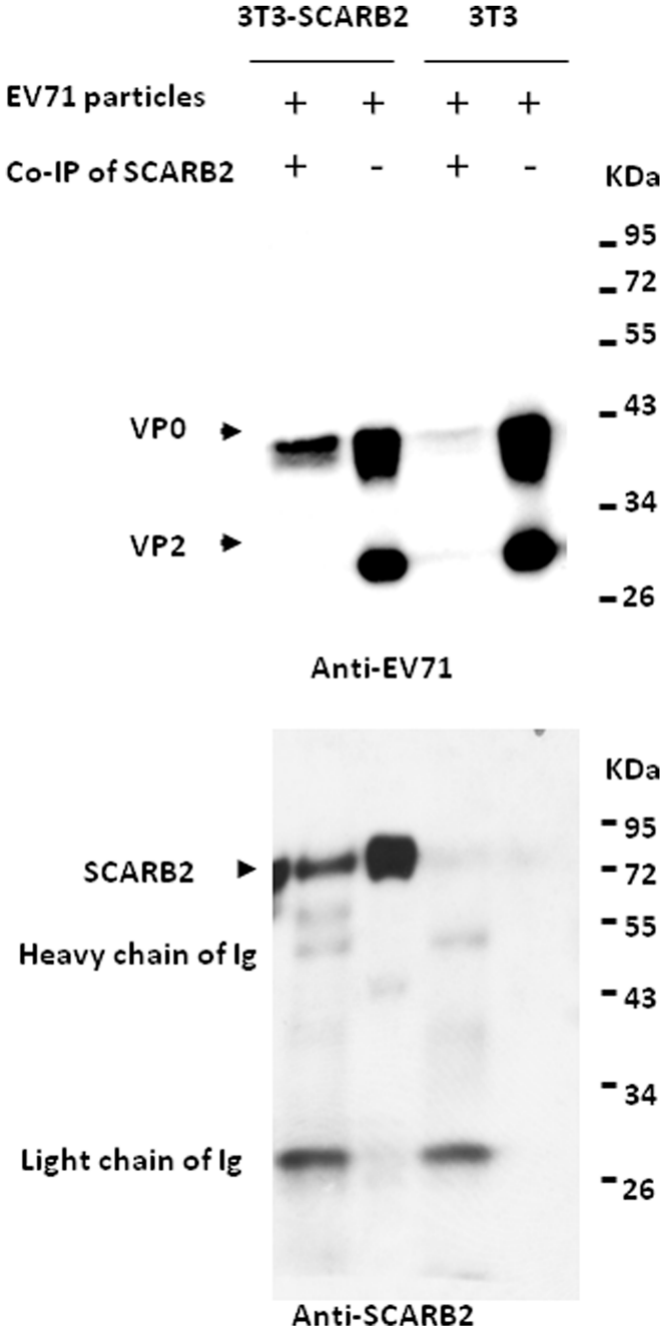
Association of EV7 with SCARB2 in 3T3-SCARB2 cells. Lysates prepared from the 3T3-SCARB2 and NIH3T3 cells were mixed individually with EV71 particles and then precipitated by protein G-conjugated anti-SCARB2 antibody as described in the Materials and Methods. Lysate-viruses mixtures without treatment of co-immunoprecipitation were also prepared. Following the co-precipitates and lysate-virus mixtures were individually subjected to western blot against associated EV71 capsid protein by blotting with MAB979 antibody (the top panel). The same membrane was stripped and re-blotted with ant-SCARB2 antibody.

To examine whether SCARB2 interacts with EV71 in the cells, co-immunoprecipitation of EV71 virions with SCARB2 in the 3T3-SCARB2 protein was performed. Cell extracts prepared from 3T3-SCARB2 or NIH3T3 cell line was mixed with excess EV71 viral particles to allow the virus to associate with SCARB2. The complex was then immunoprecipitated using protein G-conjugated anti-SCARB2 antibody. Western blotting of precipitates with anti-EV71 MAB979 demonstrated that EV71 virions were precipitated from 3T3-SCARB2 cells but not from control NIH3T3 cells (the top panel of Fig. 3), compared to immunoblotting of lysate-viruses mixtures from both cell lines that corresponding EV71 capsid protein were observed. SCARB2 was detected in co-immunoprecitates and lysate-viruses mixtures from 3T3-SCARB2 but not NIH3T3 cells while the same membrane was stripped and then re-blotted with anti-SCARB2 antibody (the bottom panel of Fig. 3), confirming that SCARB2 interacting with EV71 capsid protein could be pulled down from 3T3-SCARB2 but not from NIH3T3 cells. This result demonstrates that SCARB2 does associate with EV71 and therefore acts as a cellular receptor for EV71 infection.

### Knocking down SCARB2 inhibits EV71 infection

**Figure 4:**
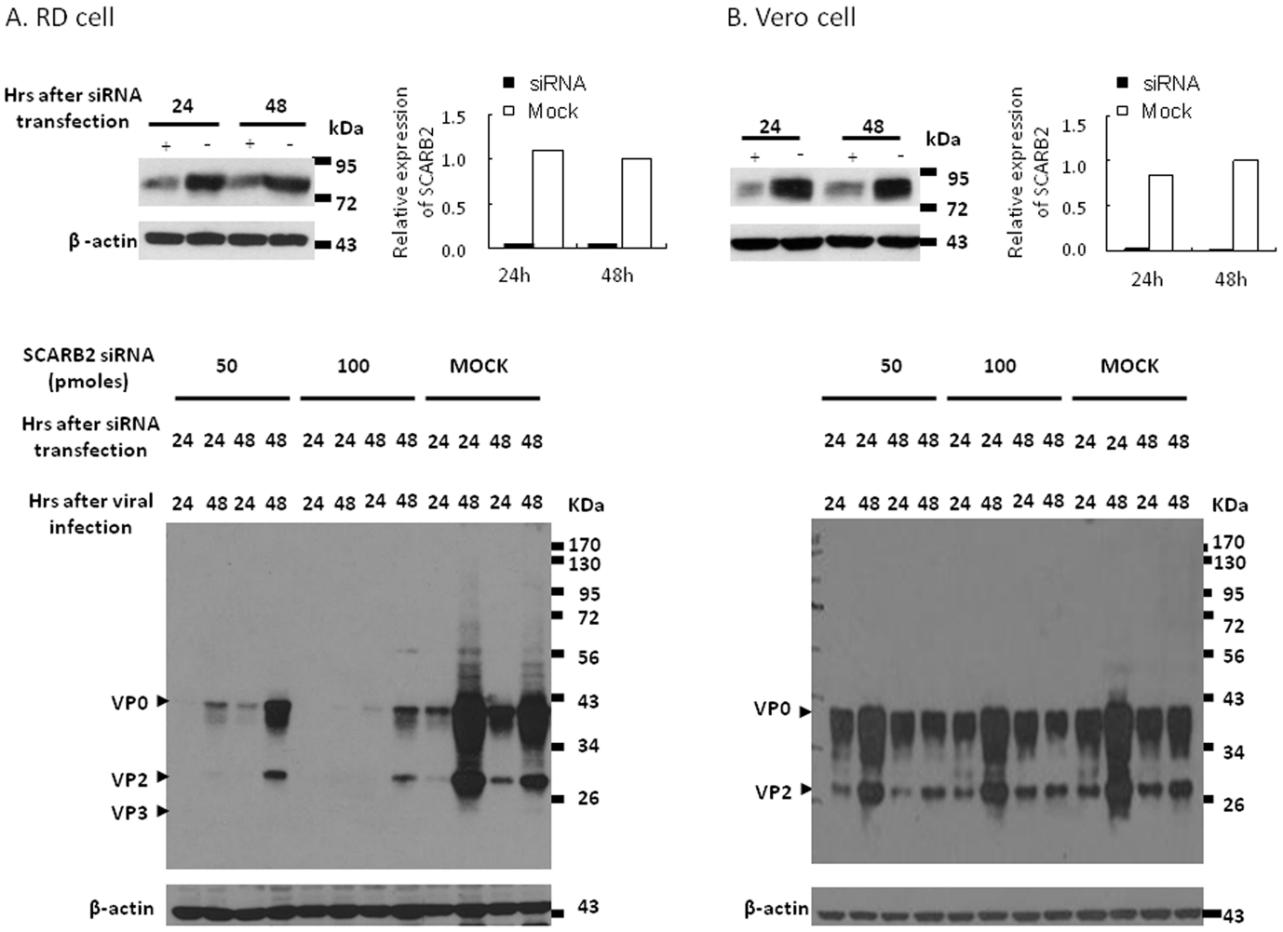
Infection of EV71 in RD cells but not Vero cells is SCARB2-dependent. (A) RD cells, and (B) Vero cells were transfected with two different amount of SCARB2 siRNA and then incubated for 24 or 48 hours before MOI = 0.04 of EV71 infection. Lysates prepared at 24 or 48 hours after siRNA transfection were subjected to western blot using anti-SCARB2 antibody (the tope panel). Cell lysates were extracted at 24 or 48 hours after EV71 infection and subjected to detect the synthetic viral capsid protein by western blot using MAB979 antibody (the lower panel). The internal control of cellular β-actin was detected by blotting the same membrane with monoclonal anti-β-actin antibody. Corresponding changes in SCARB2 protein levels compared to the expression levels in 48 hours transfection of the cells with 100 pmoles control siRNA (Mock) as 1.0 were quantified using Image-Pro Plus 6.0 software. Data represent one of two independent experiments.

To further prove that SCARB2 is the key protein supporting EV71 infection, endogenous SCARB2 expressed in RD and Vero cells was knocked down using SCARB2-specific siRNA. Cells were transfected with SCARB2 siRNA followed by 24 or 48 hours of incubation (t24 or t48), and subsequently infected with EV71 (MOI = 0.04) for 24 or 48 hours (i24 or i48). SCARB2 expression level in siRNA-transfected cells was monitored at 24 or 48 hours after transfection by Western blot using anti-SCARB2 antibody. It was found that expression of SCARB2 was reduced by almost 100% in RD cells transfected with 100 pmoles of SCARB2 siRNA, compared to control siRNA-transfected cells (the top panel of Fig. 4A). However, the expression of EV71 capsid protein in infected RD cells was completely inhibited upon transfection with 100 pmoles of SCARB2 siRNA, while the capsid protein expression was significantly reduced in cells transfected with 50 pmoles of siRNA (the bottom panel of Fig. 4A). Expression of capsid protein was not affected in mock-transfected cells.

A similar experiment performed in Vero cells revealed that transfection with 50 or 100 pmoles of SCARB2 siRNA had no inhibitory effect on the synthesis of EV71 capsid proteins (the bottom panel of Fig. 4B), even though the ∼100% of SCARB2 expression was inhibited by siRNA transfection (the top panel of Fig. 4B). These findings suggest that SCARB2 is the major receptor for EV71 infection in RD cells but not in Vero cells, where other unidentified proteins function as receptor for EV71 infection.

### Cellular entry of EV71 is dependent on clathrin and dynamin

**Figure 5:**
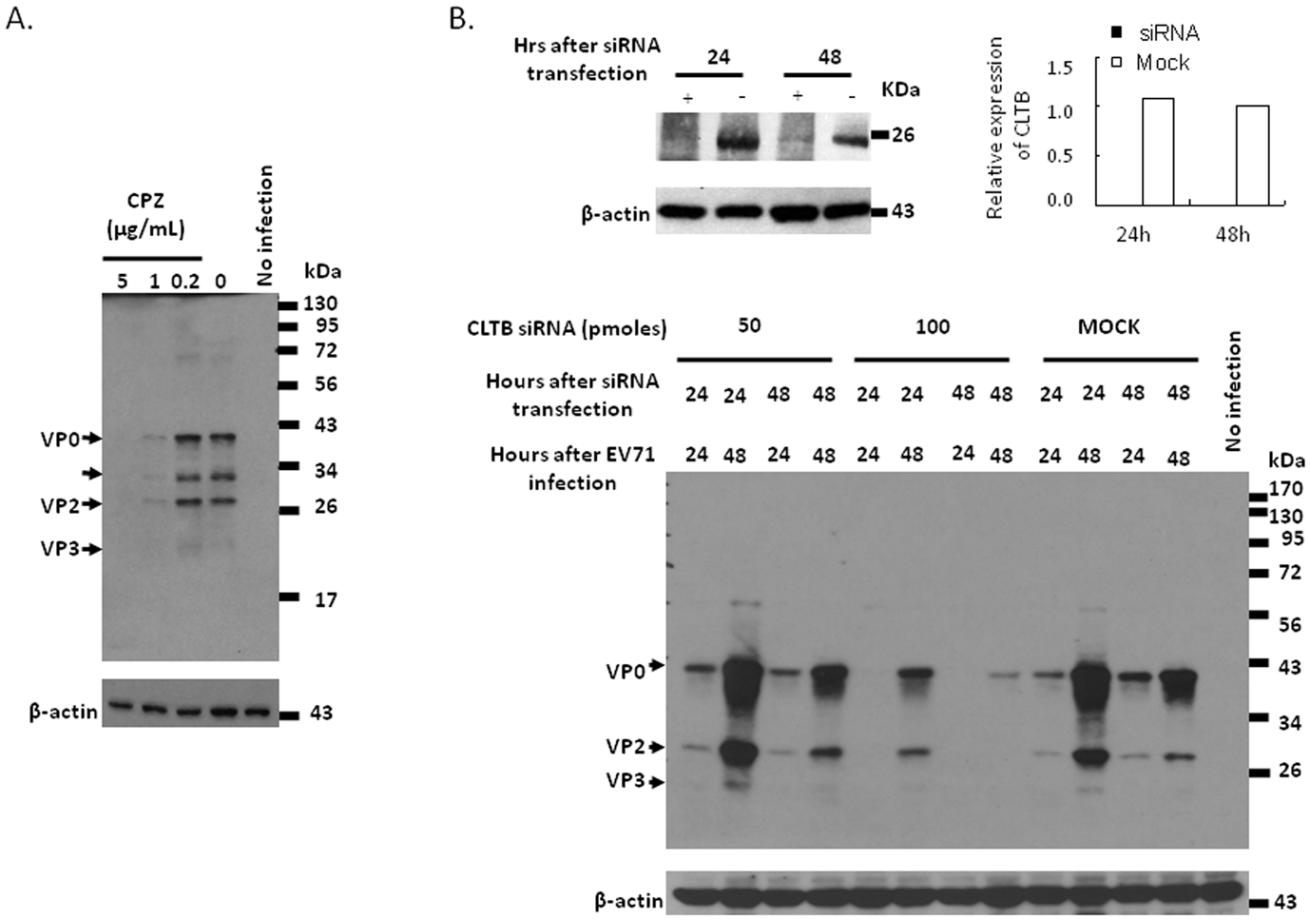
EV71 infection is impacted by clathrin specific inhibitors. (A) 3T3-SCARB2 cells were pretreated with different doses of CPZ for one hour prior to the addition of MOI = 0.04 of EV71. (B) 50 or 100 pmoles of siRNA specific to clathrin or 100 pmoles of control siRNA (MOCK) were transfected to 3T3-SCARB2 cells as following the treatment protocol as Figure 4 described above. After 24 or 48 hours of transfection, cells were following lysed and detected the content of clathrin light chain B (CLTB) by Western blot using anti-clathrin antibody. Relative expression of siRNA-targeting genes shown in the top panel represented the relative CLTB protein levels of the cells transfected with specific siRNA, compared to the expression in 48 hours transfection of the cells with 100 pmoles control siRNA (Mock) as 1.0 was shown. Lysates were also prepared after 24 or 48 hours of EV71 infection and subjected to analyze the expression of synthetic EV71 capsid protein by western blot using MAB979 antibody. The result was shown in the lower panel. Cellular β-actin was detected by blotting the same membrane with monoclonal anti-β-actin antibody. Data represent one of two independent experiments.

SCARB2 is known to induce clathrin-, but not caveolae-, mediated endocytosis in the internalization of HCV into target cells [51], and in HDL endocytosis [23]. In an attempt to unravel the mechanism of EV71 entry into 3T3-SCARB2 cells, several compounds known to inhibit clathrin- and caveolin-mediated endocytosis were used to annotate the pathway involved in EV71 entry. Chlorpromazine (CPZ) is a cationic amphiphilic reagent that inhibits the assembly of clathrin-coated pits at the plasma membrane [52], [53]. It has been used to test out clathrin-mediated endocytosis in many viruses such as influenza virus [25], HCV [26], SARS-CoV [29], VSV [30], and RSV [27]. 3T3-SCARB2 cells pretreated with various concentrations of CPZ showed a dose-dependent reduction in the synthesis of *de novo* capsid proteins following EV71 infection. Significant inhibition of capsid protein synthesis was observed at CPZ concentration of 5.0 µg/mL (Fig. 5A). These findings suggest that clathrin-mediated endocytosis played a crucial role in EV71 entry.

To further examine EV71 entry is clathrin-dependent course, endogenous clathrin expression in 3T3-SCARB2 cells was knocked down using clathrin light chain B (CLTB)-specific siRNA. Downregulation of cellular 30 KDa of CLTB expression was observed at 24 and 48 hours after transfection with 100 pmoles of clathrin-specific siRNA, compared to mock-transfected cells (the top panel of Fig. 5B). It was observed that 100 pmoles of clathrin siRNA was sufficient to block EV71 capsid protein synthesis at 48 h post transfection (the bottom panel of Fig. 5B). These results prove that EV71 entry into 3T3-SCARB2 cells is facilitated by clathrin-mediated endocytosis.

**Figure 6:**
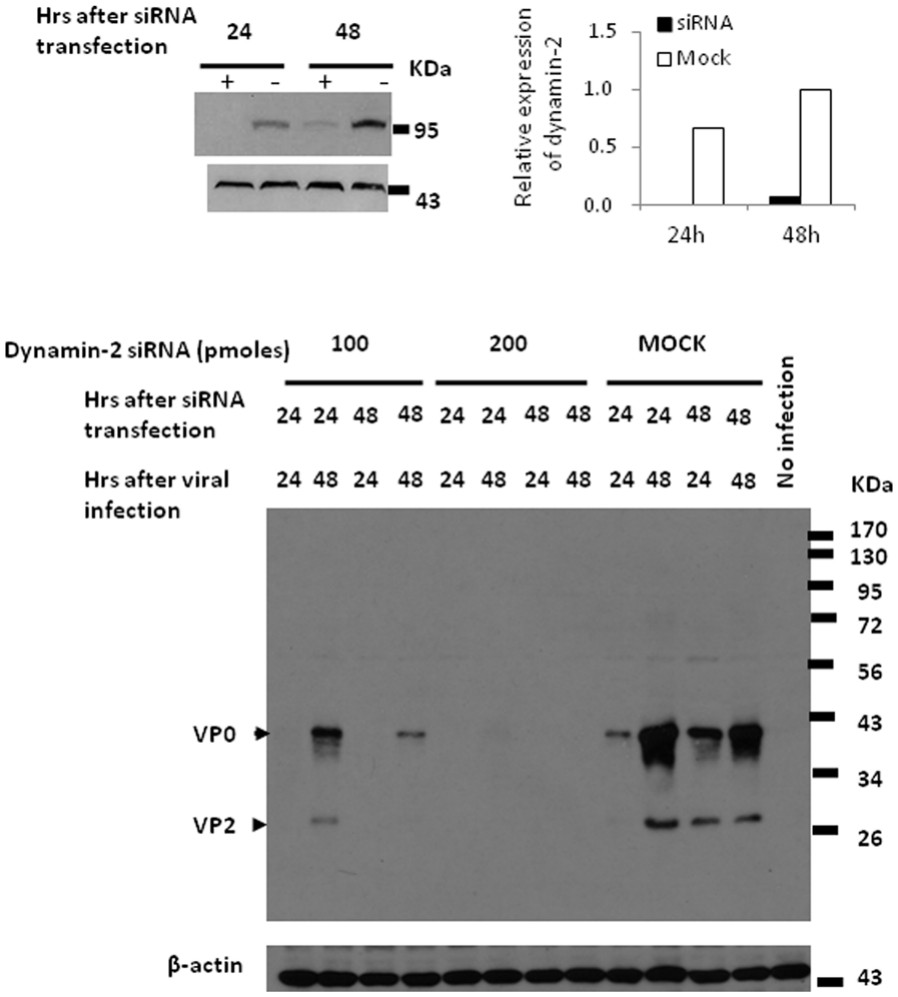
EV71 infection is blocked by dynamin-2 specific siRNA. 3T3-SCARB2 cells were treated with 100 and 200 pmoles of dynamin-2 siRNA or 200 pmole of control siRNA (MOCK) prior to the infection of EV71 as following the treatment protocol as Figure 4 described above. After 24 or 48 hours of transfection, the detection of dynamin-2 by western blot using anti-dynamin antibody was conducted, or following the transfectants were infected with MOI = 0.04 of EV71 and incubated for 24 or 48 hours before lysate preparation. Expression of dynamin-2 in the lysates was shown in the top panel. Relative dynamin-2 protein levels in specific siRNA-treated cells compared to the levels in 48 hours of cells transfected with 200 pmoles control siRNA (Mock) as 1.0 was shown. The lower panel showed the expression of EV71 capsid protein which was detected by western blotting of MAB979 antibody. Cellular β-actin was detected by blotting the same membrane with monoclonal anti-β-actin antibody. Data represent one of two independent experiments.

Dynamin family proteins have been reported to tabulate and save membrane-coated vesicles and plays a role in membrane fission that associated with clathrin-dependent and clathrin-independent endocytic processes [38], [39], [54]. The expression of dominant-negative dynamin mutants (dynamin^K44A^) has been shown to block the formation of clathrin-coated pits and vesicles [55], [56]. These observations have prompted us to investigate whether dynamin may also play a role in EV71 entry mechanism. Dynamin-2 expression could be inhibited after 24 and 48 hours of 3T3-SCARB2 cells transfected with 200 pmoles of dynamin-2-specific siRNA as judged by the detection of dynamin (∼100 KDa) in the immunoblot assay (the top panel of Fig. 6). Dynamin-2-specific siRNA pretreatment of 3T3-SCARB2 cells affected the EV71 virus replication in a dose-dependent manner. The production of viral capsid proteins was completely inhibited in cells transfected with 200 pmoles of dynamin-2 siRNA, whereas only moderate inhibition of viral capsid protein was seen in cells treated with 100 pmoles of dynamin-2 siRNA (Fig. 6). Our observations confirm that dynamin plays a crucial role in EV71 endocytosis through clathrin-mediated pathway.

### EV71 entry is independent of caveolae

**Figure 7:**
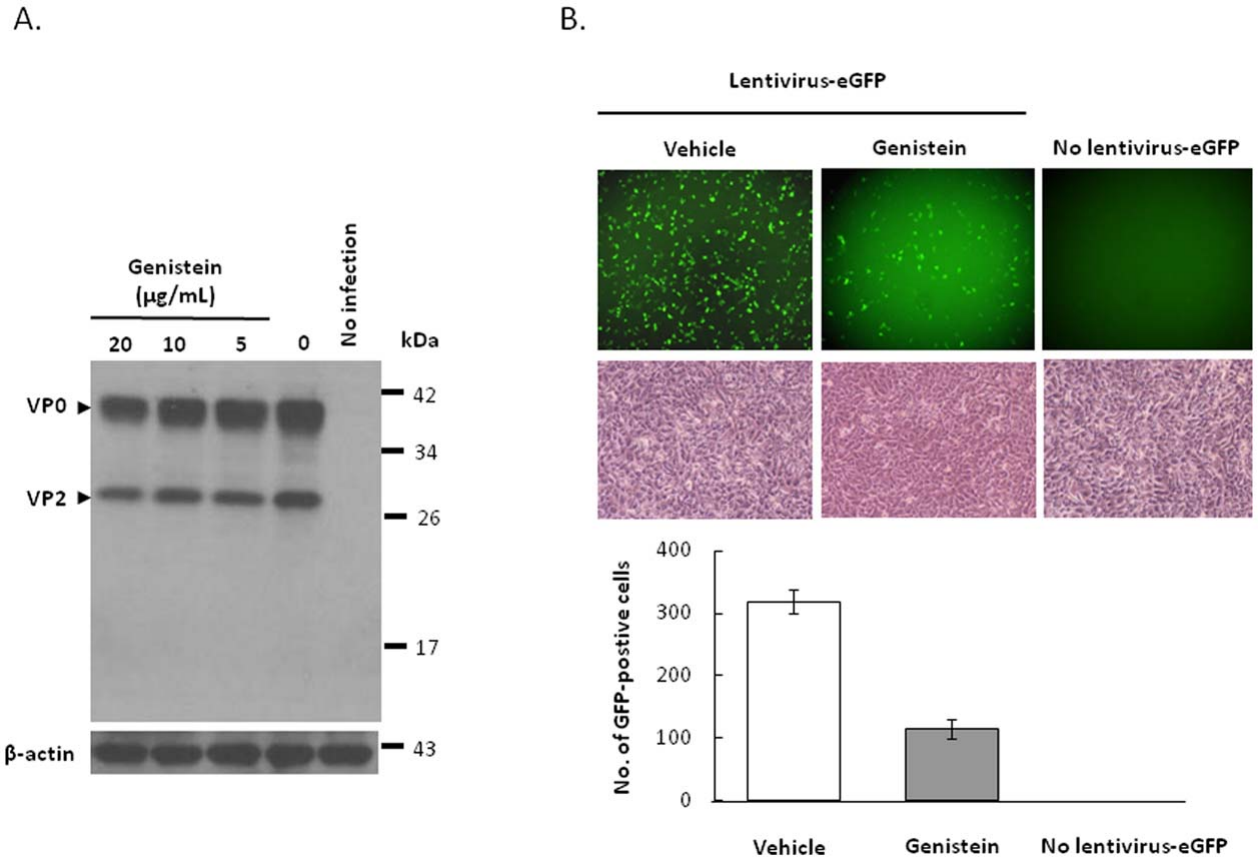
EV71 entry was not affected by a tyrosine kinase inhibitor treatment. (A) Pretreatment of 3T3-SCARB2 cells with 20, 10, and 5 µg/mL of genestin for one hour before MOI = 0.04 of EV71 infection was conducted. After one hour infection, the cells were incubated for 24 hours. Cells lysate were further prepared and subjected to western blot using MAB979 antibody to detect EV71 capsid protein. The internal cellular β-actin was detected by blotting the same membrane with monoclonal anti-β-actin antibody. (B) As a positive control of genistein inhibitory effect, A549 cells were pre-treated with vehicle or 20 µg/mL of genistein for one hour before infected with MOI = 0.1 of lentivirus-eGFP, and then following the cell culture until 72 hours incubation. Cells without lentivirus-eGFP infection as negative control were also included. The same field of fluorescent and visible pictures was taken under the *UV*-fluorescent microscopy and GFP-positive cells were counted.

**Figure 8:**
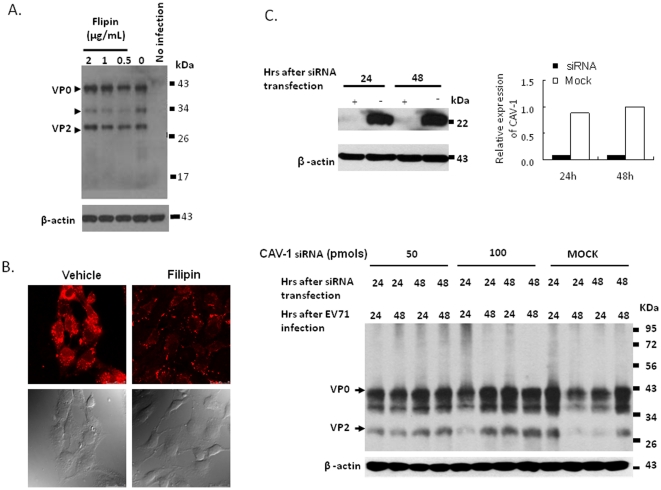
Caveolae-independence of EV71 entry. (A) Pretreatment of 3T3-SCARB2 cells with 2. 1, and 0.5 µg/mL of filipin was conducted as the same condition as the legend of Fig. 7A described above. Cells lysates were analyzed by western blot using MAB979 antibody to detect EV71 capsid protein. The internal cellular β-actin was also detected by blotting the same membrane. (B) Uptake of 5 µg/mL CT-B conjugated with alexa fluor 594 in 3T3-SCARB2 cells treated with 2 µg/mL of filipin or vehicle (0.1% DMSO) was conducted following the protocol described in the Materials and Methods. The same field of fluorescent and visible pictures was taken under confocal microscopy. (C) Transfection of 3T3-SCARB2 cells with 100 pmoles of caveolin (CAV-1) siRNA or control siRNA or control siRNA (Mock) prior to the infection of EV71 as following the treatment protocol as Figure 4 described above was conducted. Expression of CAV-1 in the lysates was shown in the top panel. Relative expression of CAV-1 in specific siRNA-treated cells compared to the protein levels in 48 hours of cells transfected with 100 pmoles control siRNA (Mock) as 1.0 was shown. The lower panel showed the expression of EV71 capsid protein which was detected by western blotting of MAB979 antibody. Cellular β-actin was detected by blotting the same membrane with monoclonal anti-β-actin antibody. Data represent one of two independent experiments.

Besides clathrin-dependent endocytosis, other alternate mechanisms have been described to be involved in viral entry. Caveolae have been implicated in the uptake of viruses such as SV40, polyomavirus, ebola virus, echovirus I, lentivirus, and others [28], [31], [32], [33], [34], [57]. In the study of caveolae-mediated virus entry, genistein, a tyrosine kinase inhibitor, can interrupt caveolae-mediated endocytosis [58], [59], [60], and filipin, a cholesterol-binding agent, is capable of disrupting the caveolar structure and function [61], [62], [63], were subjected to test the ability in the inhibition of EV71 infection using a two-fold of the maximally effective dose that has been reported [58], [61], [62]. 3T3-SCARB2 cells were treated with genistein prior to the infection with EV71. Maximal dose of 20 µg/mL genistein had no inhibitory effect on the endocytosis of EV71 in infected 3T3-SCARB2 cells (Fig. 7A), as compared to the control group of A549 cells which pre-treated with the same dose of genistein was significantly resistant to lentivirus-eGFP infection (Fig. 7B). Similar result was observed in 3T3-SCARB2 cells pre-treated with the maximal dose of 2 µg/mL filipin that had no inhibition in EV71 entry into 3T3-SCARB2 cells (Fig. 8A). It was in contrast to the significant suppression of the uptake of alexa fluor 594 cholera toxin subunit B (CT-B) conjugate in 3T3-SCARB2 cells by the same dose of filipin (Fig. 8B). The mechanism of cholera toxin internalization through caveolae endocytosis has been confirmed [62]. Caveolin riched in NIH3T3 cells was known [64]. We further knocked down the endogenous caveolin-1 (CAV-1) by specific siRNA. Caveolin expression could be inhibited after 24 and 48 hours of 3T3-SCARB2 cells transfected with 100 pmoles of CAV-1 siRNA as judged by immunoblot of the caveolin (22 KDa) (the top panel of Fig. 8C). However, 3T3-SCARB2 cells pre-treated with CAV-1 siRNA did not resist from EV71 infection (Fig. 8C). These results show clearly that EV71 entry using SCARB2 receptor is through clathrin- but not caveolae-dependent endocytosis.

### EV71 endocytosis is dependent on endosomal pH and intact membrane cholesterol

Generally, enveloped viruses attach to the host cells through bind to the receptor on the surface of host and then enter either at acidic pH or at neutral pH. Acidic pH-dependent group includes VSV, influenza A virus, adenovirus and rubella virus [65]. Some viruses like RSV, SV40, measles virus, herpes simplex virus I, vaccinia virus and HIV-1 are insensitive to lysosomotropic agents [27], [65], [66], [67] which are able to block endosomal acidification. For most viruses using clathrin-mediated endocytosis, transition from early-to-late stage endosome is pH-dependent [65]. After endocytosis, viruses containing early endosmoes are configured to form acidic (pH 6.0 to 6.5) vesicles and become progressively more acidic as they mature to form late endosomes (pH 5.5 to 6.0). Progressive acidification of endosomes is required for the internalized viruses to establish their infection [65], [68].

**Figure 9:**
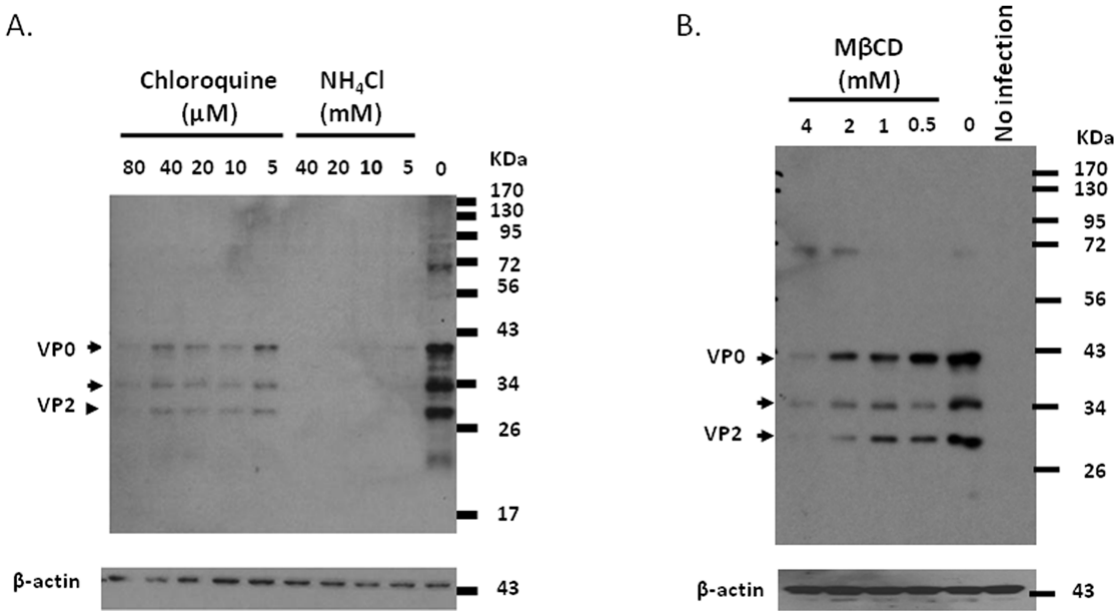
EV71 endocytosis is endosomal pH dependent and the intact membrane cholesterol dependent. 3T3-SCARB2 cells were pretreated with various doses of (A) chloroquine and NH_4_Cl, or (B) methyl β-cyclodextrin (MβCD), for one hour prior infection with MOI = 0.04 of EV71. After 24 hours incubation in the presence of drugs, cells lysate were prepared and subjected to western blot using MAB979 antibody to detect synthetic EV71 capsid protein. The internal cellular β-actin was detected by blotting the same membrane with monoclonal anti-β-actin antibody.

Specific compounds like NH_4_Cl [27], [60] and chloroquine [26], [27], [60] are capable of inhibiting endosomal acidification and therefore employed to verify whether this phenomenon is consequential for EV71 infection. 3T3-SCARB2 cells were treated with varied concentrations of NH_4_Cl or chloroquine prior to EV71 infection. It was observed that NH_4_Cl was able to inhibit EV71 capsid protein synthesis at concentration as low as 5 mM, while chloroquine showed a dose-dependent inhibition at the concentration of 5 µM with the maximal effect seen at 80 µM compared to untreated cells (Fig. 9A). These observations confirm that endocytosis of EV71 is pH dependent.

Cholesterol is required for receptor-mediated endocytosis of viruses *via* CCPs [69] and is essential for the structure and function of invaginated caveolae and caveolae-dependent endocytosis [70], [71], [72]. Both clathrin- and caveolae-mediated endocytosis require intact membrane cholesterol and are perturbed by methyl β-cyclodextrin (MβCD), an agent that removes cholesterol from the plasma membrane [73]. Pretreatment of 3T3-SCARB2 cells with various concentrations of MβCD prior to infection with EV71 revealed that MβCD was capable of inhibiting capsid protein synthesis in a dose-dependent manner (Fig. 9B). Viability of 3T3-SCARB2 cells was almost 100% at MβCD concentration of 4 mM or less, but cytotoxicity was observed at MβCD concentration of 8 mM (data not shown). Our results indicate that intact cell membrane cholesterol is essential for EV71 entry by clathrin-mediated endocytosis.

Our observations demonstrate that the route of internalization of EV71 particles in SCARB2-expressing cells involves clathrin, dynamin and is dependent on endosomal acidification and membrane cholesterol level.

## Discussion

Productive infection of target cells by viruses requires entry pathways that allow viral components to be introduced into the cytoplasm for subsequent processes such as uncoating, gene expression, and replication. Previous studies with EV71 indicate that SCARB2 [17] and PSGL-1 [16] serve as cellular receptors for EV71 infection. Although endogenous mouse SCARB2 expressed by NIH3T3 cells has 85.8% homology to human SCARB2, it seems not to participate in the acute infection of NIH3T3 cells with EV71. Only 3T3-SCARB2 cells that overexpress human SCARB2 were highly susceptible to EV71, confirming that the expressed human SCARB2 serves as a receptor conferring susceptibility to EV71 infection. We have also observed that NIH3T3 cells cultured with EV71 for a long time (>5 days), showed some non-specific infection and replication of the virus, as indicated by the cytopathic effect and plaque formation (data not shown). This might be due to either mouse SCARB2 or some other cell surface protein functioning as a receptor for EV71, albeit with very low specificity. Mouse SCARB2 is a poor receptor for EV71 infection of mouse cells was also confirmed by Yamayoshi et al. [74]. In addition, Vero cells that are used as hosts for EV71 infection and production [16], [75] have marginal expression of SCARB2 but no expression of PSGL-1 confirming earlier observation [16]. It is possible that the apparently low expression of SCARB2 in Vero cells might be due to low affinity of the experimental-used anti-SCARB2 polyclonal antibody towards green monkey SCARB2. Additionally, siRNA specific to SCARB2 could not inhibit EV71 infection in Vero cells even though the expression level of SCARB2 was impaired (Fig. 5), indicating that in these cells some other protein functions as a receptor for EV71 entry.

The interaction between SCARB2 receptor and EV71 capsid protein in 3T3-SCARB2 cells infected with EV71 has been clearly demonstrated by co-immunoprecipitation. In addition to ensure the role of SCARB2 as a receptor for EV71, our study also reveals that EV71 enters cells through clathrin- and dynamin-dependent endocytosis with progressive acidification of the endosomes. Similar pathways have been reported to be employed by other viruses. Polio virus uses CD155, a member of the immunoglobulin superfamily as a receptor to infect host cells [76], [77], [78] and enters the cells independent of clathrin and endosomal acidification [79]. RSV enters the cells through clathrin-mediated endocytosis at neutral pH [27], [80]. Influenza virus enters target cells by both clathrin-dependent and -independent pathways of endocytosis [81], [82].

It has been reported that upon endocytosis, the native viral particles containing the genomic viral RNA form subviral “A-particles” sedimenting at 135S, which then undergo a conformational change to create subviral “B-particles” that sediment at 80S induced by the low pH (≤5.6) at the endocytic carrier vesicles and late endosomes. Release of viral RNA from the protein shell of “B-particles” facilitates viral replication [55], [83], [84]. This antigenic conversion mediated by low pH is decreased in potassium-depleted cells [55]. Therefore, the resulting acidification of the endosomal environment would have enhanced the conversion of EV71 “B-particles” to release the viral RNA resulting in enhanced expression of viral capsid proteins in the endosomal stage of EV71 infection.

In this study we confirm the role of SCARB2 as a receptor for EV71 and determine its internalization pathway to involve clathrin, dynamin and progressive acidification of endosomes. Our study paves way for understanding EV71 infectious pathway in human SCARB2 expressing cells and therefore is of consequence in planning prophylactic as well as therapeutic approaches to control EV71 infection.
